# Thienoisoindigo-Based Semiconductor Nanowires Assembled with 2-Bromobenzaldehyde via Both Halogen and Chalcogen Bonding

**DOI:** 10.1038/s41598-018-32486-z

**Published:** 2018-09-27

**Authors:** Juran Noh, Sungwoo Jung, Dong Geon Koo, Gyoungsik Kim, Kyoung Soon Choi, JaeHong Park, Tae Joo Shin, Changduk Yang, Juhyun Park

**Affiliations:** 1 School of Chemical Engineering and Materials Science, Institute of Energy Converting Soft Materials, Chung-Ang University (CAU), Seoul, 06974 Republic of Korea; 2 Department of Energy Engineering, School of Energy and Chemical Engineering, Perovtronics Research Center, Low Dimensional Carbon Materials Center, Ulsan National Institute of Science and Technology (UNIST), Ulsan, 44919 Republic of Korea; 3 Advanced Nano-Surface Research Group, Korea Basic Science Institute (KBSI), Daejeon, 34133 Republic of Korea; 4 Department of Molecular Engineering, Kyoto University, Katsura, Nishikyo-ku, Kyoto, 615-8510 Japan; 5 UNIST Central Research Facilities & School of Natural Science, Ulsan National Institute of Science and Technology (UNIST), Ulsan, 44919 Republic of Korea

## Abstract

We fabricated nanowires of a conjugated oligomer and applied them to organic field-effect transistors (OFETs). The supramolecular assemblies of a thienoisoindigo-based small molecular organic semiconductor (TIIG-Bz) were prepared by co-precipitation with 2-bromobenzaldehyde (2-BBA) via a combination of halogen bonding (XB) between the bromide in 2-BBA and electron-donor groups in TIIG-Bz, and chalcogen bonding (CB) between the aldehyde in 2-BBA and sulfur in TIIG-Bz. It was found that 2-BBA could be incorporated into the conjugated planes of TIIG-Bz via XB and CB pairs, thereby increasing the π − π stacking area between the conjugated planes. As a result, the driving force for one-dimensional growth of the supramolecular assemblies via π − π stacking was significantly enhanced. TIIG-Bz/2-BBA nanowires were used to fabricate OFETs, showing significantly enhanced charge transfer mobility compared to OFETs based on pure TIIG-Bz thin films and nanowires, which demonstrates the benefit of nanowire fabrication using 2-BBA.

## Introduction

Organic transistors have been developed over the last decades as next-generation electronic devices owing to their versatility for flexible devices and facile fabrication process compared to rigid inorganic semiconductors^1,2^. In particular, one-dimensional (1D) organic nano/micro-wire transistors have attracted significant attention due to their excellent electronic properties, including high charge-carrier mobility in specific directions^3–6^. Various strategies have been used to prepare organic semiconducting wires, including an ink-jet method^7,8^, electro-spinning^9^, hard^10–12^ and soft template tools^13–15^, self-assembly in solution^16–19^ and physical vapor transport^20,21^. The excellent charge-carrier mobilities were obtained for self-assembled single crystals of organic semiconductors, because their closely assembled structures by intermolecular interactions enable electrons to move efficiently through a conjugated backbone plane^16,22^.

Organic semiconductors are prone to be crystallized due to the presence of π − π stacking of the conjugated main backbones, which provides the driving force for 1D growth. In general, the self-assembly of single crystalline based on nano/micro-wires of the organic semiconductors shows the strong dependence of the solubility on recrystallization temperature^17,19,23^ and solvent type^18,21,24^, which is usually determined by the molecular structure of the organic semiconductors^16,25,26^. Organic semiconductors usually require flexible alkyl side chains to facilitate solution processing^27^. However, the alkyl side chains increase the tendency for lateral packing of the organic semiconductors and disturb 1D growth in the direction of the π − π stacking of conjugated planes during precipitation or recrystallization, resulting in spherical morphologies due to hydrophobic interactions in the lateral direction of the conjugated planes^28^. Therefore, enhancing the driving force for π − π stacking so that π − π interactions between conjugated planes dominate lateral hydrophobic interactions between alkyl chains is critical for preparing nano/micro-organic semiconductor wires.

Halogen bonding (XB) is a strong and tunable form of non-covalent bonding between halogen atoms and negative sites, with bonding strengths of 10–150 kJ/mol^29^. Since the XB originates from the attraction between the partially positive charged region of halogen atoms, the σ hole, and its counterpart in electron-rich donor atoms, it has been widely used in supra-molecular assembly and crystal engineering of self-assembled structures^30–35^. The halogen atoms have dipolar charges; the π hole is perpendicularly negative, while the σ hole is horizontally positive. These charges simultaneously attract electron-rich functional groups, including lone-pair electrons in the same plane, and electron-poor groups in the vertical direction^36^. Furthermore, the XB should benefit for electronic device applications because it does not involve acidic or basic groups that can trap charges and disturb charge transfer, which is clearly different to hydrogen bonding^37^. Hence, the XB is considered an important intermolecular interaction for co-crystallization with conjugated oligomers or polymers for organic electronic devices^38–45^.

Chalcogen bonding (CB) is a newly identified type of weak non-covalent interaction, recently described by using various modern characterization techniques, such as nuclear magnetic resonance, X-ray photoelectron spectroscopy (XPS), and X-ray diffraction (XRD). Despite its weak interaction, the CB can play a dominant role in crystal design as it has a controllable binding strength^46–48^ of a few to hundreds of kJ/mol^49^. The CB involves highly polarizable and electronegative atoms (i.e., chalcogen atoms (O, S, Se, Te) which have σ holes and π holes similar to halogen atoms. In this way, the σ hole, the positive part of chalcogen atoms at the opposite side of the covalent bond, can combine with the electron donor and bind via electrostatic interaction^50^. In most chalcogen-containing organic semiconductors, the CB plays an important role in facilitating charge transport by the improving electronic delocalization of their backbones^41,50^.

Although it has been proven that CB or XB is a useful interaction for molecular assembly, most approaches using CB or XB for transistor devices based on organic semiconductors have focused on intramolecular interactions in planar conjugated planes and consequently high carrier mobility^38–45,50–52^. Semiconducting oligomers or polymers bearing chalcogen or halogen atoms can have enhanced intramolecular electron delocalization and narrow optical band gaps due to the planar structure of their backbones. For example, the thienoisoindigo (TIIG) unit has been used as a promising building block to construct high-mobility organic semiconductors for organic field-effect transistors (OFETs). Advantages of this material include strong π − π stacking with a large overlapping area induced by a combination of the planar backbone structure and intramolecular CB, where improved electronic delocalization is derived from the quinoidal structure^51,52^. At present, it is hard to find molecular assemblies of organic semiconductors utilizing intermolecular XB and CB.

In this study, we fabricated nanowire assemblies of a TIIG-based organic semiconductor, (E)-2,2′-diphenyl-4,4′-bis(2-ethylhexyl)-[6,6′-bithieno[3,2-b]pyrrolylidene]- 5,5′(4 H,4 H′)-dione, TIIG-ethylhexyl benzene (TIIG-Bz), which was able to simultaneously engage in XB and CB interactions with 2-bromobenzealdehyde (2-BBA), and demonstrated their use for organic field-effect transistor (OFET) applications. We examined the TIIG-Bz/2-BBA nanowire assemblies using XPS, powder XRD (PXRD), grazing incidence XRD (GIXD), and XPS, and investigated the influence of 2-BBA on nanowire assemblies and charge transfer mobility of OFETs.

## Results and Discussion

**Figure 1:**
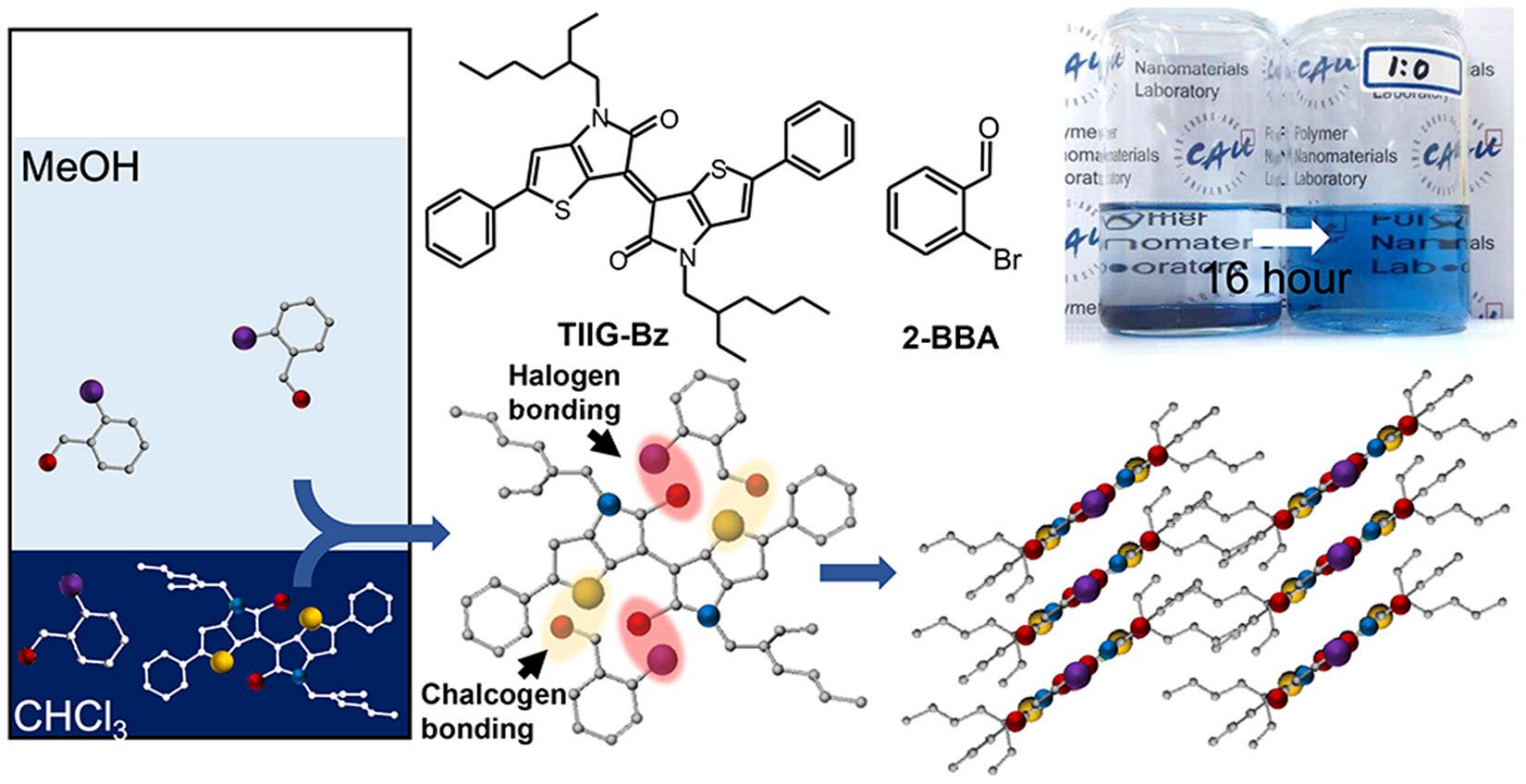
Schematic illustrations of the chemical structures of TIIG-Bz and 2-BBA and their packing motif assembled via a bisolvent phase transfer method.

Nanowires of TIIG-Bz and supra-molecular nanowire assemblies of TIIG-Bz and 2-BBA were fabricated using the bisolvent phase transfer method^28,32,53^, shown schematically in Fig. 1. This method is a popular fabrication process for nano-wire assemblies because it optimally accommodates molecules with different structures and sizes. In the process, a solution with organic semiconductors dissolved in a good solvent is placed in a vial, then a poor solvent is carefully poured onto the solution. Despite quite good miscibility between the poor and good solvents, their density difference kept them from immediately mixing with each other. Instead, at the interface of the two miscible solvents, conjugated organic molecules dissolved in the good solvent slowly self-assemble as the two solvents mix and the solubility of the organic semiconductor decreases. Consequently, self-assembled nanowires are formed at the interface^28,32,53^. In this study, for supra-molecular assemblies, TIIG-Bz and 2-BBA were dissolved in chloroform (d = 1.4459 g/cm^3^ at 20 °C), a good solvent for both chemicals, and co-precipitated at the interface between chloroform and methanol (d = 0.7914 g/cm^3^ at 20 °C) upon the inter-diffusion of chloroform and methanol.

**Figure 2:**
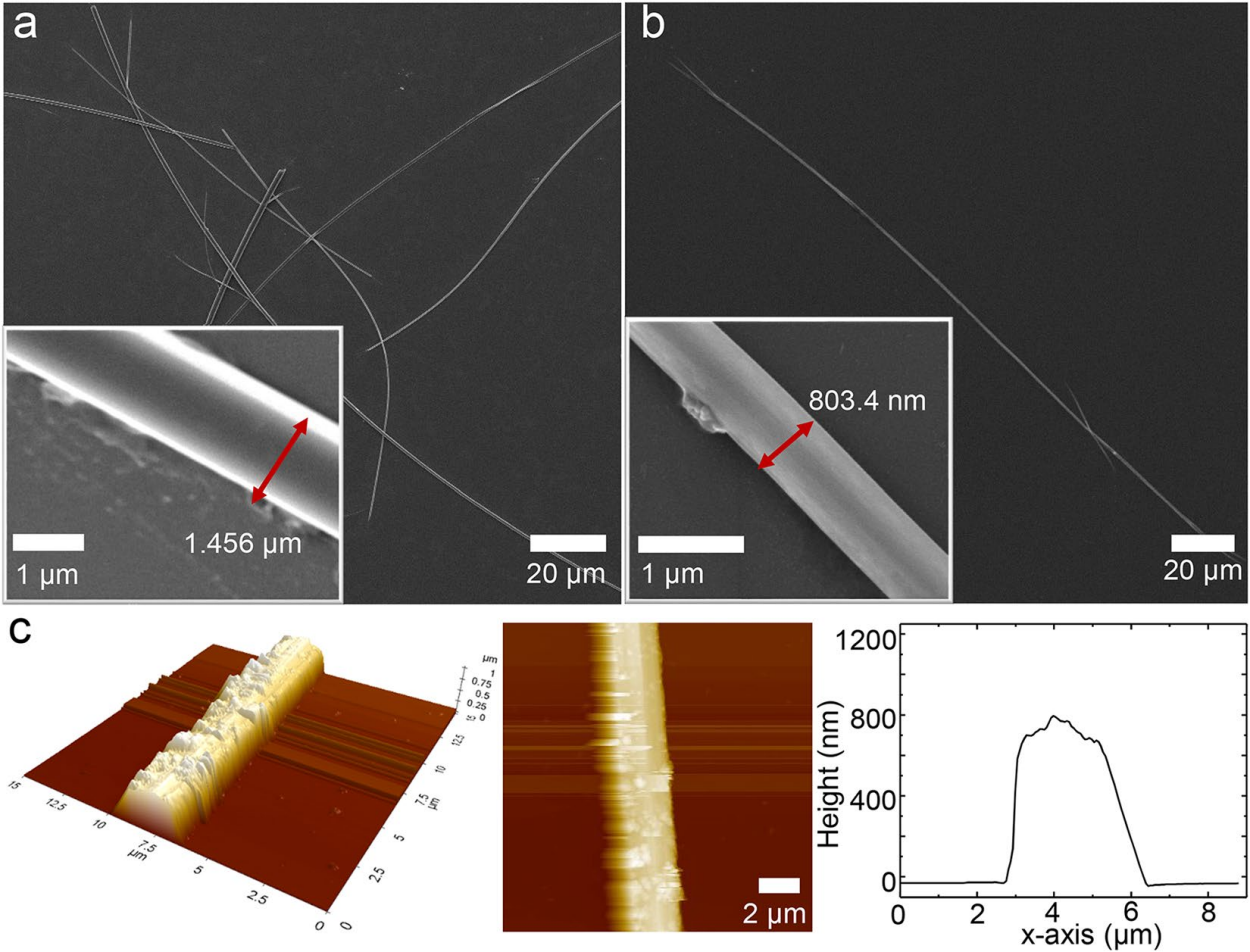
FE-SEM images of (**a**) the nascent TIIG-Bz wire assembly and (**b**) TIIG-Bz/2-BBA nanowire. (**c**) 2D and 3D AFM height profiles of the TIIG-Bz/2-BBA nanowire.

As shown in Fig. 2(a), nanowires of only TIIG-Bz with a thickness ~1–2 μm (60–200 μm in length) were formed via this bisolvent phase transfer process. The crystals of TIIG-Bz molecules preferentially grow in one direction, even without the use of 2-BBA, due to their molecular rigidity and planarity that enhance intermolecular π − π interactions upon exposure to methanol when dissolved in chloroform. However, it should be noted that some TIIG molecules were aggregated in a bundle of wires and formed much thicker wire groups or particles (Fig. S1), indicating inhomogeneous self-assembly in both planar and vertical direction of conjugated plane due to competition between π − π stacking of conjugated planes and lateral packing of ethylhexyl chains. When TIIG-Bz was assembled with 2-BBA via bi-phase separation, supra-molecular nanowires were successfully assembled with a higher aspect ratio, thickness ~800 nm, and length ~180 µm, than the TIIG-Bz nanowires assembled without 2-BBA, as shown in Fig. 2(b,c). During fabrication of TIIG-Bz and 2-BBA, both chloroform and methanol are good solvents for 2-BBA and we used the same concentration of 2-BBA in both the chloroform and methanol phases to prevent interphase diffusive mass transfer of 2-BBA due to concentration differences. Thus, the diffusive mass transfer of TIIG-Bz from the chloroform phase into the methanol phase was induced by a TIIG-Bz concentration gradient and enabled the formation of supra-molecular assemblies at the interface between the chloroform and methanol phases with intermixing of the solvents, followed by the decreased solubility of TIIG-Bz.

**Figure 3:**
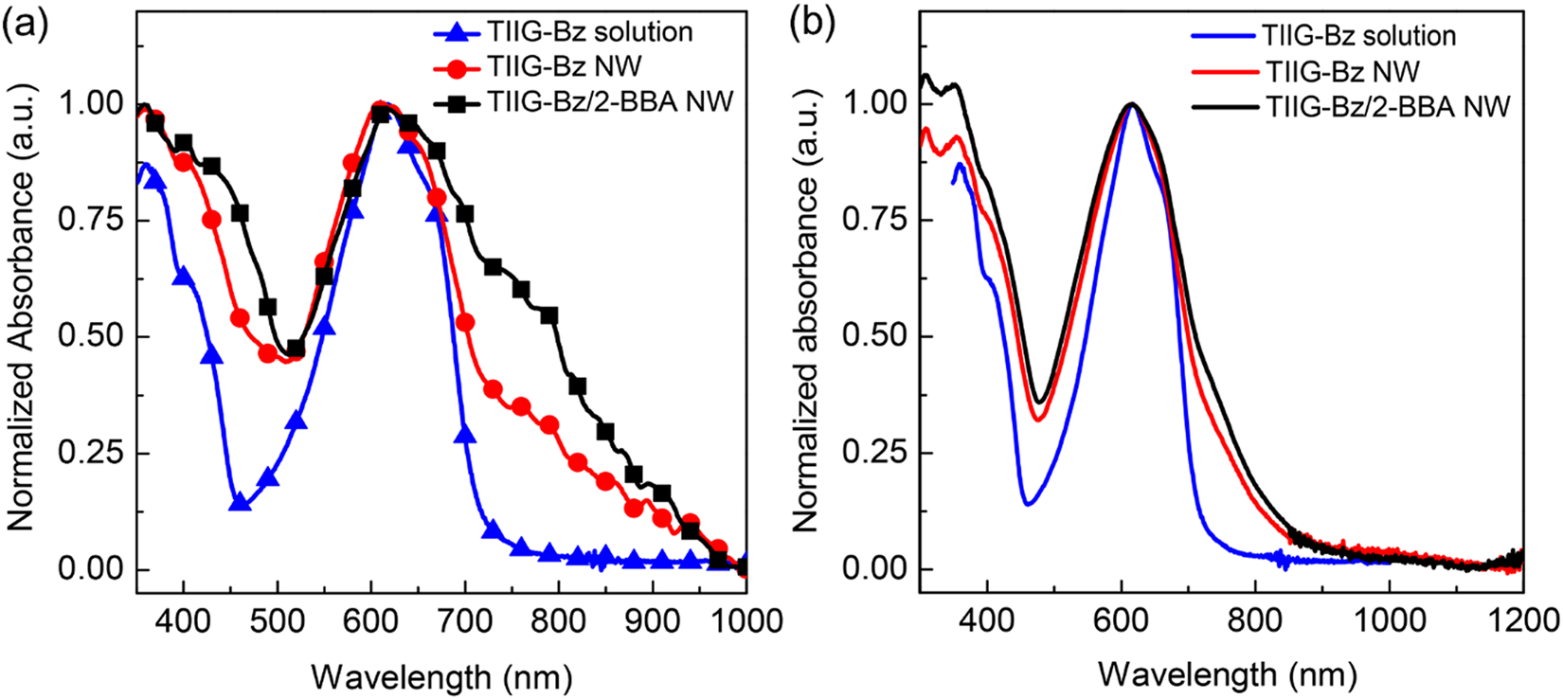
UV-Vis-NIR absorbance spectra of TIIG-Bz chloroform solution (blue line) and nanowire (red line) and TIIG-Bz/2-BBA nanowire (black line) dispersed in methanol, measured (**a**) without and (**b**) with an integrating sphere.

The optical properties of TIIG-Bz dissolved in chloroform and its nanowires dispersed in methanol were examined by UV-Vis-NIR spectroscopy. When TIIG-Bz was dissolved in the chloroform solution, the maximum absorption band appeared at 616 nm with a shoulder at around 650 nm (Fig. 3). In comparison, the absorption spectra of TIIG-Bz assemblies with and without 2-BBA dispersed in methanol showed increasing absorption intensity in the NIR region of 700–1000 nm. The maximum absorption peaks of the TIIG-Bz solution, TIIG-Bz-only, and TIIG-Bz/2-BBA nanowires were all observed at ~616 nm. However, the shoulder band around 700 nm in the spectrum of the TIIG-Bz solution was significantly red-shifted to near 800 nm for the assembled intermolecular structure as shown in Fig. 3(a). In addition, the absorption intensity of the shoulder band in the TIIG-Bz/2-BBA assembly spectra for wires were strongly enhanced compared to those of the pristine TIIG-Bz samples. Such absorbance enhancement of the TIIG-Bz/2-BBA in the NIR region of the spectra was clearly shown even after excluding any scattering effect by measuring the spectra with an integrating sphere. The higher absorbance of the TIIG-Bz wires assembled with 2-BBA in the NIR region was consistent with our recent results of density functional theory calculations for conjugated polymer nanoparticles^54,55^. Such a red shift in the absorption spectra originates from enhanced intermolecular π − π interactions with energy level adjustments, and has been widely observed in thin films for organic optoelectronic devices or nanomaterials of conjugated polymers^56–61^. Furthermore, this enhancement in NIR absorption clearly indicated improved intermolecular π − π interactions in the direction of nanowire growth, which was significantly enhanced with increasing nanowire length, showing better aggregation.

**Figure 4:**
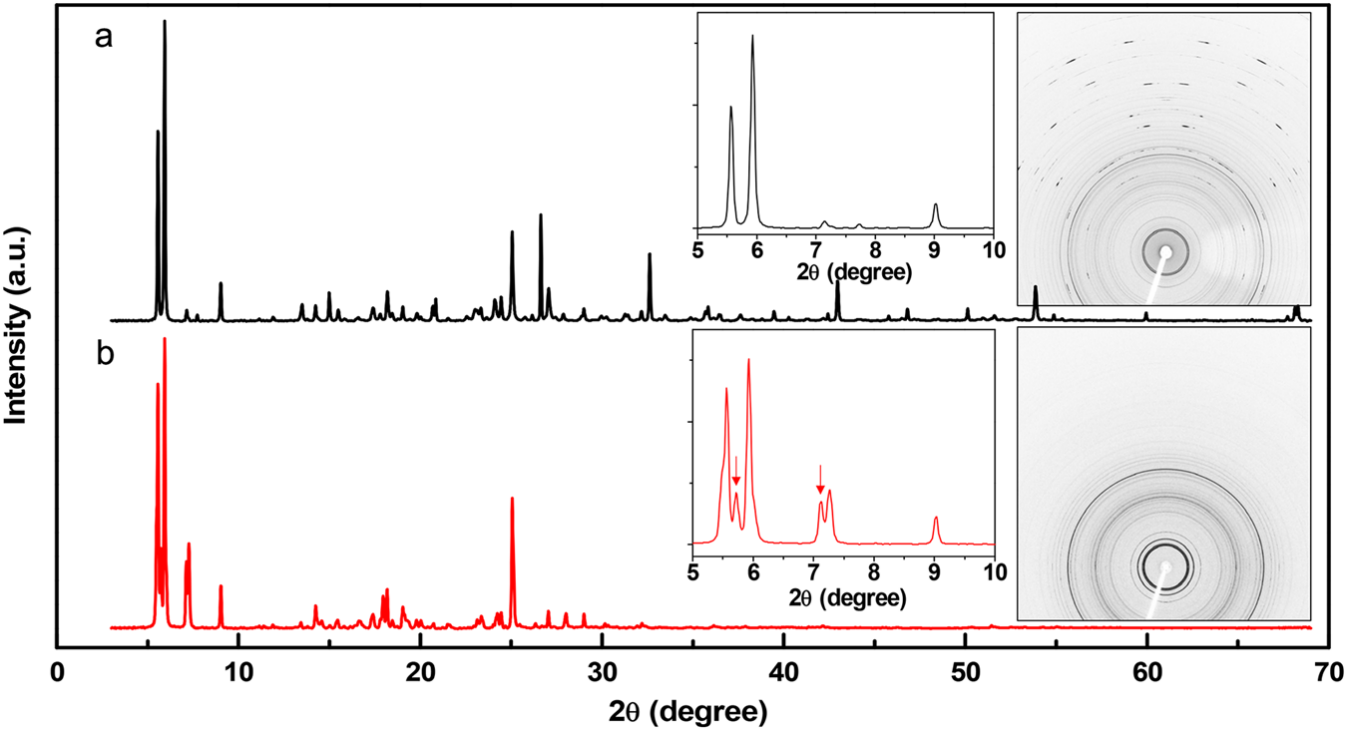
PXRD images and profiles of (**a**) TIIG-Bz nanomaterial and (**b**) TIIG-Bz/2-BBA nanowires.

Molecular assembly structures of TIIG-Bz and TIIG-Bz/2-BBA nanowires characterized by PXRD showed clearly different diffraction patterns depending on the incorporation of 2-BBA into TIIG-Bz assemblies. The 2D PXRD pattern of TIIG-Bz nanomaterials (right inset of Fig. 4(a)) shows ring patterns in the small-angle range and short dashes in the wide-angle range up to 2θ = 70° that were symmetric to the vertical, central line as the measurements were made over 360°. These short dashes are a typical feature of single crystals. On the other hand, the TIIG-Bz/2-BBA nanowires showed powder-like features, i.e., peaks with identical intensities along the Debye ring (right inset of Fig. 4(b)). Reflecting these characteristics of the 2D PXRD patterns, the circular averaged 1D profile of the TIIG-Bz/2-BBA nanowires (Fig. 4(b)) showed weakened peaks in the wide-angle range above 2θ = 30°, while that of TIIG-Bz nanomaterials showed peaks up to 2θ = 70° (Fig. 4(a)). It should be noted that identification of all crystallographic peaks in our PXRD data was difficult as both the TIIG-Bz nanomaterials and TIIG-Bz/2-BBA nanowires may not have been single-phase. As shown in the SEM image of Figure S1, TIIG-Bz-only nanomaterials were a mixture of wires, needles, and particles. In addition, the TIIG-Bz/2-BBA nanowires may have been a mixture of assemblies of TIIG-Bz, TIIG-BZ and one 2-BBA, and TIIG-Bz and two 2-BBA molecules due to inhomogeneous 2-BBA distribution. Detailed peak assignment is being performed by preparing single crystals or supramolecular assemblies with homogeneous morphology (this study will be published separately). However, at present, it is obvious that a new structure is developed upon the addition of 2-BBA into the TIIG-Bz assembly, as shown in the left inset of Fig. 4. In the inset 1D PXRD pattern (below 2θ = 10°), TIIG-Bz nanomaterials showed five diffraction peaks at 2θ = 5.563°, 5.933°, 7.121°, 7.734°, and 9.023°. In comparison, new diffraction peaks (indicated by the arrows) were obvious in the 1D PXRD pattern of TIIG-Bz/2-BBA nanowires, where six diffraction peaks appeared at 2θ = 5.563°, 5.716°, 5.933°, 7.146°, 7.261°, and 9.023° (Figure S2). The diffraction peaks of both TIIG-Bz and TIIG-Bz/2-BBA assemblies were assigned to a triclinic lattice structure (Figure S2; a = 11.75, b = 15.48, c = 16.47 Å, α = 101.504, β = 98.13, and γ = 99.44° for TIIG-Bz assembly; and a = 13.53, b = 17.52, c = 18.74 Å, α = 111.29, β = 108.22, and γ = 99.95° for TIIG-Bz/2-BBA nanowires). It is worth noting that the lattice parameters and full-width-at-half-maximum values of the diffraction peaks (Table S1) increased with the addition of 2-BBA into the TIIG-Bz assembly. The PXRD results indicated that 2-BBA molecules in TIIG-Bz/2-BBA nanowires limit the growth of TIIG-Bz-only crystals and make the crystal structure of the original TIIG-Bz assembly a bit larger. As shown in the SEM image in Fig. 3(b) and (c), it seems that the addition of 2-BBA is advantageous for 1D nanowire growth of TIIG-Bz assembly.

**Figure 5:**
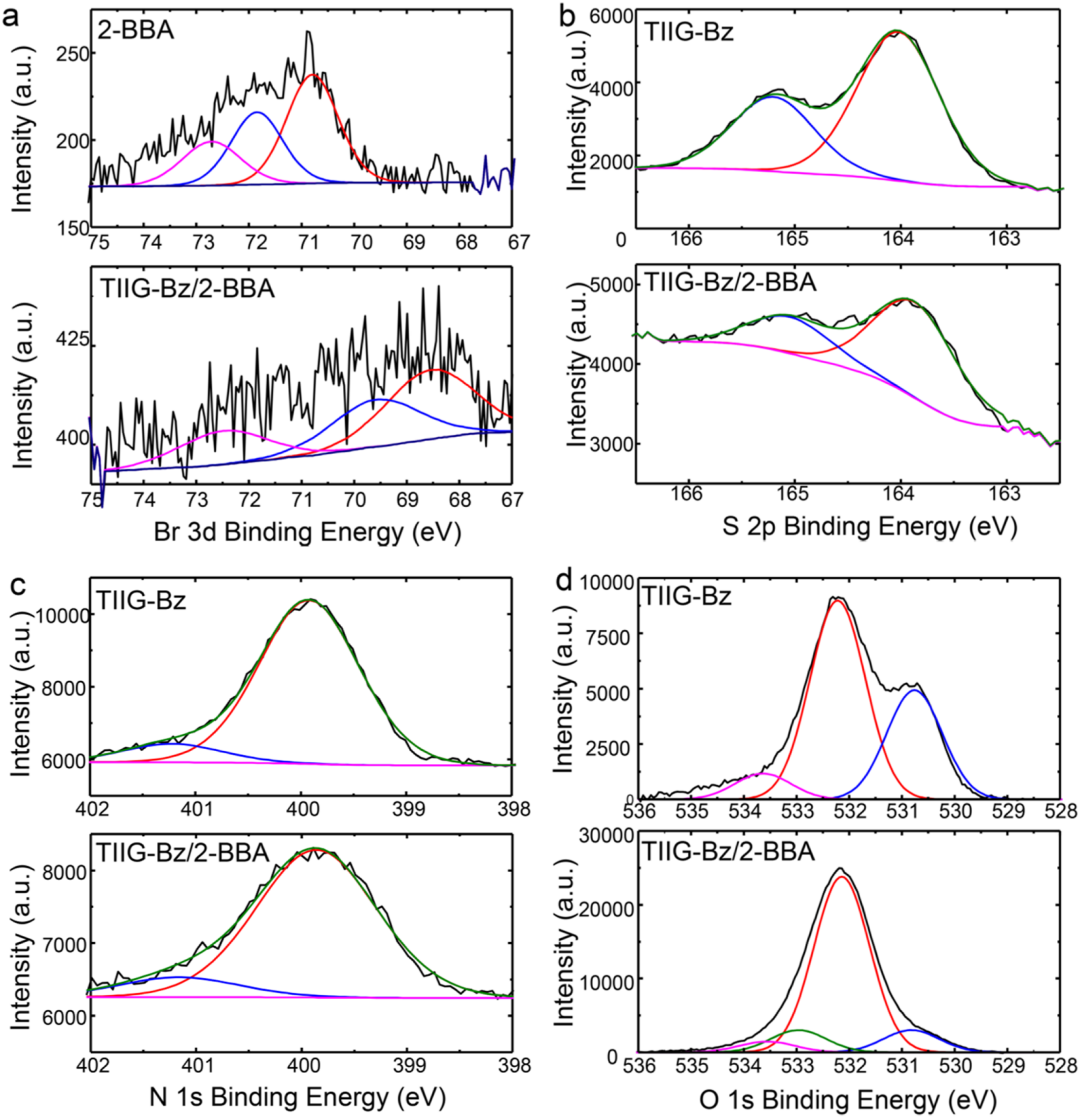
XPS spectra of (**a**) Br 3d in 2-BBA, and TIIG-Bz/2-BBA nanowires, (**b**) S 2p, (**c**) N 1 s, and (**d**) O 1 s in TIIG-Bz, and TIIG-Bz/2-BBA nanowires.

The existence of both XB and CB in TIIG-Bz/2-BBA nanowires was confirmed by analyzing XPS spectra, as shown in Fig. 5. The changes of the binding energies in the XPS spectra for each atom and compound are summarized in Table 1. XB and CB are intermolecular interactions between an electron donor and an electron accepter that partially give and take electron pairs. Bromine or chalcogen atoms that have the electron-deficient σ hole can act as the electron accepter in this interaction; these elements can be bound to electron-rich oxygen in carbonyl groups and partially receive electrons via XB and CB. As a result, the electrons of these elements are increased and the binding energies of each electron become weaker. The decrease in the binding energy of the bromine atom in 2-BBA was observed upon assembly of 2-BBA with TIIG-Bz, as shown in Fig. 5(a). The binding energy of bromine 3d_5/2_ in 2-BBA was 70.86 eV and that of the TIIG-Bz/2-BBA nanowires decreased by 1.60 eV to 69.26 eV, which is a characteristic of XB^62,63^. In addition, the binding energies of the chalcogen atom, sulfur in TIIG-Bz, 2p_3/2_, and 2p_1/2_ decreased from 164.02 and 165.20 to 163.94 and 165.12 eV by 0.08 eV, respectively, as shown in Fig. 5b. The larger change in binding energies of bromine compared to sulfur was due to the stronger binding of XB than CB as the σ hole of the bromine atom is more positive than that of sulfur; this is consistent with a recent report related to the XB mechanism^62^.

**Table 1:** Binding energies of Br 3d, S 2p, and O 1 s in 2-BBA, TIIG-Bz, and TIIG-Bz/2-BBA nanowires.

Atom	Compound type	2-BBA [eV]	TIIG-Bz [eV]	TIIG-Bz/2-BBA [eV]	Δ Binding Energy [eV]
Bromine	3d 5/2	70.86		69.26	−1.60
Sulfur	2p 3/2		164.02	163.94	−0.08
	2p 1/2		165.20	165.12	−0.08
Oxygen	1 s aromatic-C=O		530.76	530.84	+0.08
	1 s aliphatic-C=O	532.5		532.96	+0.46

^a^The parameters of the OFETs and ONWTs were obtained by measuring 6–10 devices.

^b^Composition: molar ratio of TIIG-Bz to 2-BBA.

On the other hand, the binding energies of N 1 s in both TIIG-Bz and TIIG-Bz/2-BBA nanowires were the same (399 eV), as shown in Fig. 5(c). This result is explained as follows. It was shown previously that the electron donor of XB and CB is usually a non-covalent electron pair of nitrogen^30,64,65^. However, in TIIG-Bz, the non-covalent electrons of nitrogen are delocalized in the thienoisoindigo moieties and are oriented perpendicular to the conjugated plane. In addition, ethyl hexyl chains sterically hinder the close association of the nitrogen atom with 2-BBA. Hence, the nitrogen in TIIG-Bz is unable to bind with electron acceptors via XB or CB. Instead of nitrogen, electron-rich oxygen in the aromatic carbonyl of TIIG-Bz can contribute to XB or CB as electron donors. However, it was difficult to confirm the effect of the addition of 2-BBA in the TIIG-Bz composite using the O 1 s binding energy spectrum due to the presence of multiple external peaks, including methanol or water (~534 eV), carbon dioxide (~532 eV), and the Si substrate (~532 eV). Figure 5(d) shows peaks at 530.76 eV that were attributed to contributions from O 1 s electrons in the aromatic carbonyls of TIIG-Bz. The strongest peak for both TIIG-Bz and TIIG-Bz/2-BBA nanowires was at 532.14 eV and was assigned to the Si-O peak from the SiO2 wafer substrates. Noticeably, the binding energies of O 1 s electrons in TIIG-Bz increased by 0.08 eV (from 530.76 eV to 530.84 eV) where their intensities significantly decreased, indicating electron donation from O 1 s electrons to XB or CB. Meanwhile, O 1 s electrons in the aliphatic carbonyl group of 2-BBA should have a binding energy at 532.5 eV^66,67^. However, their binding energy increased by 0.46 eV to 532.96 eV when 2-BBA was assembled with TIIG-Bz (Fig. 5(d)). One of the most plausible explanations for these peak changes in the O 1 s spectra is that oxygen in the aromatic carbonyl of TIIG-Bz interacts with the σ hole in bromine of 2-BBA via XB, while the other oxygen in the aliphatic carbonyl of 2-BBA reacts with the σ hole in sulfur of TIIG-Bz via CB. Thus, XPS characterization suggested that one TIIG-Bz and two 2-BBA molecules were associated with each other via both XB and CB in supramolecular assemblies of nanowires, forming slipped one-dimensional stacks^68^, as schematically illustrated in Fig. 1.

**Figure 6:**
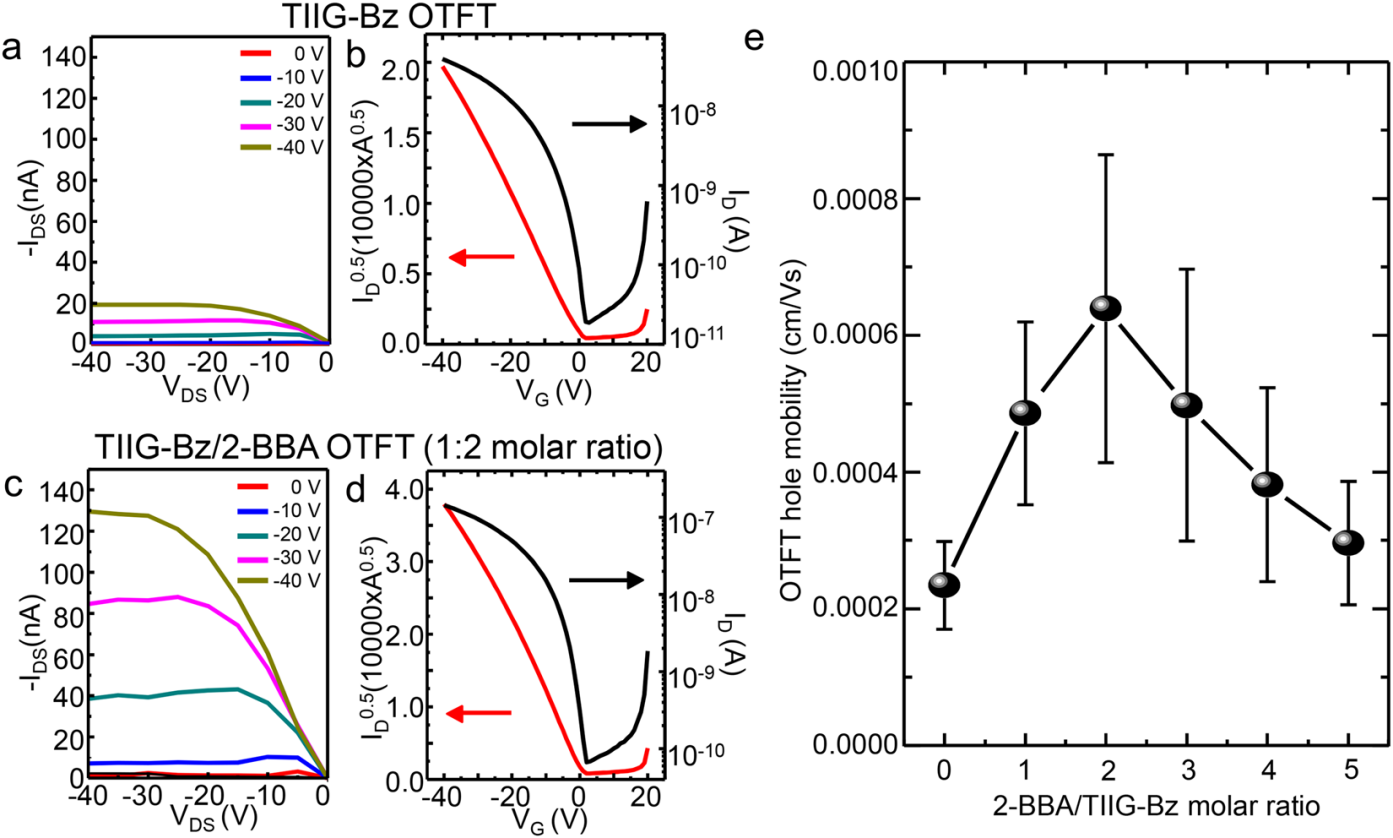
(**a**) Output and (**b**) transfer characteristics of OFET devices using thin films of TIIG-Bz, and (**c** and **d**) those of TIIG-Bz/2-BBA at a 1:2 molar mixing ratio. (**e**) Field-effect mobilities of TIIG-Bz/2-BBA thin film OFETs as a function of the molar mixing ratio of2-BBA to TIIG-Bz.

The configuration of one TIIG-Bz and two 2-BBA molecules in supramolecular assemblies (as shown in Fig. 1) was indirectly verified by measuring charge transfer efficiencies of OFETs based on thin films of TIIG-Bz and 2-BBA mixtures with various mixing ratios from 1:1 to 1:5. The devices had a BG/TC structure. Figure 5(a) and (b) show the output and transfer curves of an OFET based on a pristine TIIG-Bz thin film with a low hole mobility of 2.34 × 10^−4^ cm^2^/Vs. In comparison, the output and transfer curves of a thin film OFET fabricated with a 1:2 molar mixing ratio of TIIG-Bz to 2-BBA (Fig. 6(c) and (d)) exhibited the highest hole mobility of 6.39 × 10^−4^ cm^2^/Vs (about three times higher when we measured the hole mobilities of the OFETs with the different mixing ratios (Fig. 6(e) and Table 2; average values from seven devices); representative transfer and output curves are shown Fig. S3. This optimal molar mixing ratio was used to fabricate thin films of TIIG-Bz and 2-BBA, which clearly indicated that the TIIG-Bz/2-BBA film was the most well-ordered and the structure with one TIIG-Bz and two 2-BBA molecules was optimum for charge carrier transfer as TIIG-Bz has two positions available for interaction with 2-BBA via XB and CB. It should be noted that the assembly structure of the pristine TIIG-Bz film was not significantly changed by mixing it with 2-BBA for spin coating the film. GIXD profiles of the pristine TIIG-Bz film and TIIG-Bz/2-BBA with a 1:2 mixing ratio (Fig. S4) showed identical diffraction peak positions, indicating no significant differences in the lattice structures. However, the profile of the TIIG-Bz/2-BBA film showed stronger ordering in the assembly structure than that of the pristine TIIG-Bz film. These results suggested that the spin-coating time was not sufficient to produce a new lattice structure by the assembly of 2-BBA with TIIG-Bz, while the addition of 2-BBA enhanced structural ordering of TIIG-Bz assembly. The enhanced ordering at the optimal ratio of 1:2 strongly supports the hypothesis that two 2-BBA molecules interacted with one TIIG-Bz molecule. We propose that simultaneous XB and CB bonding between TIIG-Bz and 2-BBA increased the overall area of the conjugated plane, resulting in enhancement of the intermolecular transfer of charge carriers via the strongly ordered molecular structure. In the case of higher 2-BBA contents (i.e., the 1:3, 1:4, and 1:5 ratios), it also seemed that unbounded 2-BBA acted as an impurity on the TIIG-Bz/2-BBA complex and interfered with the formation of well-ordered complexes, resulting in reduced charge transfer mobility.

**Table 2:** Device characteristics of OFETs based on thin films and nanowires of TIIG-Bz and TIIG-Bz/2-BBA.

Device^a^	Chemical	Composition^b^	W/L [μm/μm]	μ_sat_ [cm^2^/Vs]	I_on_/I_off_	V_th_ [V]
Thin film	TIIG-Bz		1500/150	2.34 × 10^−4^	2.64 × 10^3^	−7.32–2.14
	TIIG-Bz/2-BBA	1:1	1500/150	4.86 × 10^−4^	4.53 × 10^3^	0.06–0. 57
		1:2	1500/150	6.39 × 10^−4^	1.47 × 10^3^	0.19–4.46
		1:3	1500/150	4.98 × 10^−4^	3.75 × 10^3^	5.10–5.60
		1:4	1500/150	3.81 × 10^−4^	6.42 × 10^2^	4.59–6.95
		1:5	1500/150	2.96 × 10^−4^	1.03 × 10^2^	5.40–11.0
Nanowires	TIIG-Bz		1–4 /100	3.50 × 10^−3^	6.24 × 10^1^	3.02–11.92
	TIIG-Bz/2-BBA		0.5–1/100	9.34 × 10^−3^	6.05 × 10^2^	−3.05–−1.18

**Figure 7:**
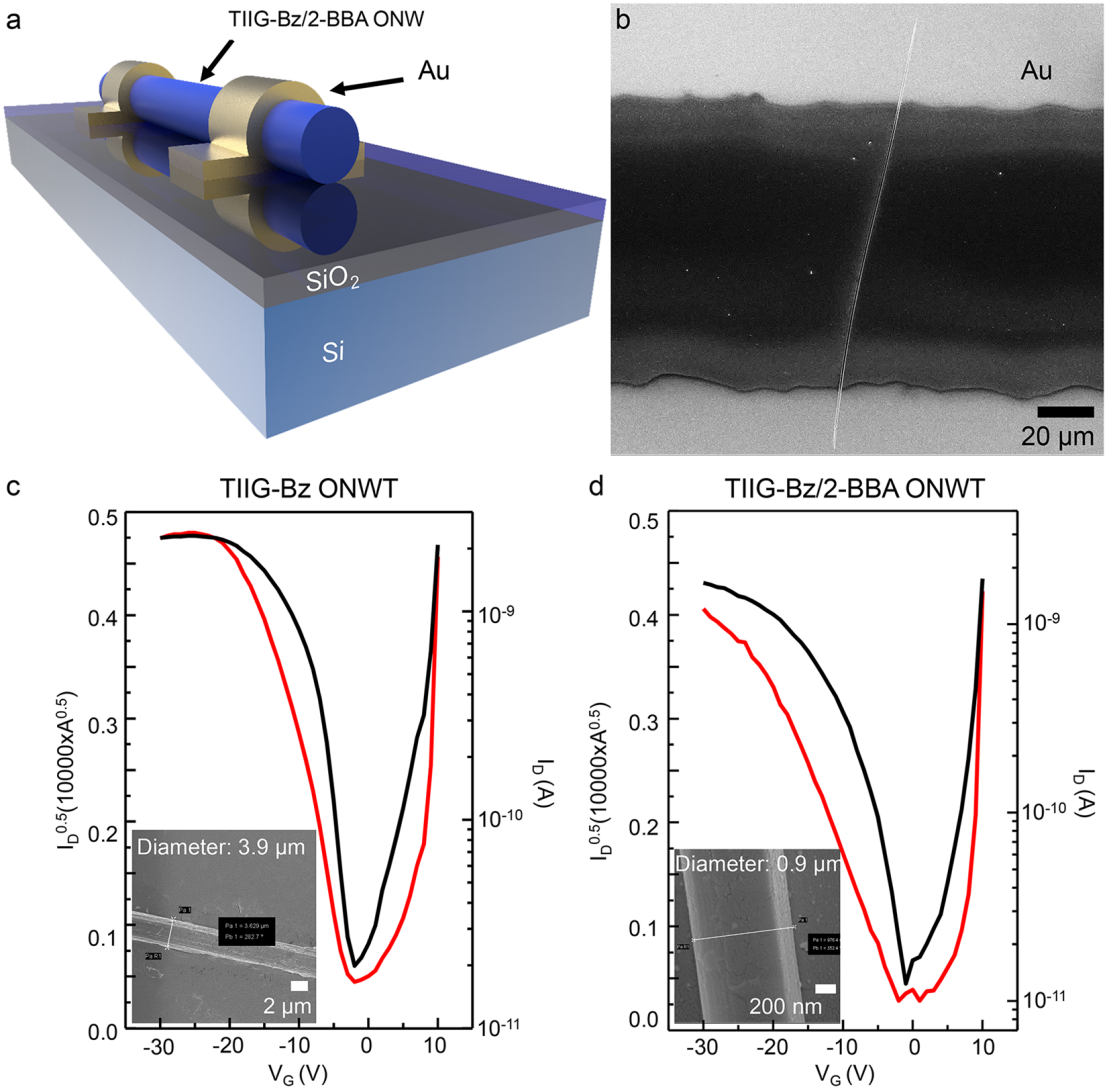
(**a**) Schematic illustration and (**b**) FE-SEM image of the organic nanowire FET device. Transfer characteristics of OFETs based on (**c**) pristine TIIG-Bz nanowires and (**d**) TIIG-Bz/2-BBA nanowires.

The applicability of supramolecular nanowire assemblies based on TIIG-Bz and 2-BBA was demonstrated by fabricating organic nanowire FETs. We fabricated OFETs using both nascent TIIG-Bz nanowires and TIIG-Bz/2-BBA nanowires, and investigated the intrinsic charge transport properties. We used the BG/TC device structure for facile fabrication, as schematically illustrated in Fig. 7(a) and shown by the SEM image in Fig. 7(b). The basic transistor parameters for the devices are summarized in Table 2. The OFET based on the TIIG-Bz nanowires showed a hole mobility of 3.50 × 10^−3^ cm^2^/Vs, about one order of magnitude higher than OFETs based on the pristine TIIG-Bz thin films (2.34 × 10^−4^ cm^2^/Vs), as shown in the representative transfer curve of the TIIG-Bz nanowire OFET device (Fig. 7(c)). The nanowires in the OFET contributed to increasing device performance by improving hole transport mobility via the nanowires compared to the thin-film-based OFET. The improved mobility was due to one-directional assembly causing the holes to move directly to the cathode, as is widely accepted^3,4,9^. The OFET based on TIIG-Bz/2-BBA nanowires showed an even higher hole mobility (9.34 × 10^−3^ cm^2^/Vs), three times higher than that of the OFET based on TIIG-Bz nanowires, as shown in Fig. 7(d) and Table 2. The highest hole transfer mobility was 0.01146 cm^2^/Vs. It should be noted that there was excess 2-BBA in the chloroform solution, ten times more than the molar content of TIIG-Bz in the preparation of TIIG-Bz/2-BBA nanowires. However, the excess 2-BBA in the nanowire preparation process did not deteriorate the device characteristics, but rather, significantly improved the mobility. This suggests that the excess 2-BBA available for dissolution in the good solvents (chloroform and methanol) during bisolvent phase transfer process was excluded from the formation of TIIG-Bz/2-BBA composite nanowires without interfering with the formation of the well-ordered supra-molecular assembly. Overall, it was confirmed that the OFET based on TIIG-Bz/2-BBA nanowires assembled via XB and CB had significantly enhanced hole mobility compared to OFETs based on thin films and nanowires of TIIG-Bz; this was due to the supramolecular structure with preferred 1D orientation resulting in efficient charge carriers transfer. It should be mentioned that the charge transfer mobilities reported here were lower than or similar to those of TIIG-Bz-based OTFTs employing a different device structure, such as top gate/bottom contact and different dielectric materials^51^. However, these preliminary results offer the possibility of further improving device performance by employing well-ordered nanowires of TIIG-Bz with enlarged conjugated plane areas via both XB and CB.

## Conclusion

We demonstrated a supramolecular nanowire assembly of a TIIG-based organic semiconductor with 2-BBA and their FET device characteristics. The analysis of XPS data strongly supported the insertion of two 2-BBA molecules into the conjugated plane of one TIIG-Bz molecule via simultaneous XB and CB between TIIG-Bz and 2-BBA. This supramolecular arrangement was further supported by XRD analysis and the fact that the highest hole mobility of OFETs based on thin films was observed for a molar ratio of TIIG-Bz to 2-BBA of 1:2. When nanowires of TIIG-Bz were assembled via a bisolvent phase transfer method, an increased conjugated plane area and the insertion of 2-BBA enhanced intermolecular π − π interactions. The enhanced π − π interactions is more dominant than lateral packing by hydrophobic interactions of alkyl chains, promoting 1D crystal growth and narrow nanowire width. It was clear that the increased conjugated area of the TIIG-Bz/2-BBA supramolecular assembly allowed efficient hole transport in the crystallization direction, although the lattice structure of the original TIIG-Bz assembly become larger with incorporation of 2-BBA. The formation of TIIG-2BBA supramolecular structure suggests a new method simultaneously using XB and CB to control the self-assembled morphology and crystalline feature of organic semiconductors with increasing intermolecular interaction, improving crystallization, and achieving high charge transport mobility in OFETs.

## Methods

### Materials

TIIG-Bz (MW = 650.94 g/mol) was synthesized using a Pd-catalyzed Suzuki coupling reaction between (E)-2,2′-dibromo-4,4′-bis(2-ethylhexyl)-[6,6′-bithieno[3,2-b]pyrrolylidene]-5,5′(4 H,4 H′)-dione and phenyl boronic acid. Full details of the synthesis and characterization were published in our previous work^52^. 2-BBA (MW = 185 g/mol) were purchased from Alfa Aesar (United Kingdom) and used as received. Chloroform and methanol were obtained from the Dae-Jung Reagent Chemical Company (South Korea).

### Preparation of TIIG-Bz and 2-BBA nanowires

TIIG-Bz and 2-BBA nanowires were prepared via a bisolvent phase transfer method, as schematically illustrated in Fig. 1, following a published procedure^28,32,53^. TIIG-Bz (1.95 mg; 3 μmol) and 2-BBA (5.55 mg; 30 μmol, 10 times the molar content of TIIG-Bz) were individually dissolved in chloroform (5 mL) in two separate vials. We placed 0.5 mL of each solution in a 70 mL vial and sonicated it for 10 min to ensure complete mixing. Then, 30 mL of a methanolic 2-BBA solution with the same 2-BBA concentration as the mixed chloroform solution (0.555 mg/mL CHCl_3_) was slowly and carefully added to the chloroform mixture containing TIIG-Bz and 2-BBA to maintain separated methanol (top) and chloroform (bottom) phases. The bi-solvent mixture was set aside for 16 h and nanowires of TIIG-Bz and 2-BBA self-assembled at the interface during diffusive mixing of the two solvents due to low solubility of TIIG-Bz. The prepared nanorods were filtered through a 0.2 μm membrane and re-dispersed in methanol to remove any residual 2-BBA.

### Device fabrication

The organic nanowire FETs were fabricated by spreading 0.5 mL of the nanowire methanol solution over an n-doped Si/SiO_2_ substrate. Shadow masks yielding a channel width of 1500 μm and channel length 100 μm were overlaid on the nanowires in the vertical direction of the channel and gold electrodes with a thickness of 140 nm were deposited to complete the bottom-gate top-contact (BG/TC) devices. For comparison, BG/TC OFETs based on thin films of only TIIG-Bz and TIIG-Bz/2-BBA mixtures were fabricated by spin casting (1500 rpm, 30 s, ramp-up speed: 0.1 sec) a 2.5 mg/mL TIIG-Bz chlorobenzene solution and TIIG-Bz/2-BBA chlorobenzene solutions with different mixing ratios onto Si/SiO_2_ substrates. For a fixed amount of TIIG-Bz (2.5 mg/mL), five different 2-BBA contents were mixed at molar ratios from 1:1, 1:2, 1:3, 1:4 and 1:5 (0.71, 1.42, 2.13, 2.84, and 3.55 mg/mL of 2-BBA, respectively). The TIIG-Bz films were then annealed at 80 °C for 20 min, followed by deposition of 70-nm-thick gold electrodes via thermal evaporation through a metal shadow mask, yielding a channel length and width of 150 μm and 1500 μm, respectively. The field-effect mobility was determined in the saturation regime using the following relationship.

$${\mu }_{sat}=(2{I}_{DS}L)/(WC{({V}_{G}-{V}_{th})}^{2})$$

where *I*_*DS*_ is the saturation drain current, *L* is the channel length, *W* is the nanowire width, *C* is the capacitance (~35 nF/cm^2^) of the SiO_2_ dielectric (100 nm), *V*_*G*_ is the gate bias, and *V*_*th*_ is the threshold voltage. The device performance was evaluated in air using an HP4156A Precision Semiconductor Parameter Analyzer (Agilent, U.S.A)^69–71^.

### Characterization

The optical properties of TIIG-Bz and its nanowire assemblies with 2-BBA were examined with an ultraviolet-visible (UV-Vis) spectrometer (V-670, JASCO, USA). The UV-vis-NIR absorption spectra were also obtained using a JASCO V-670 UV-vis-NIR spectrophotometer equipped with a 60 mm BaSO_4_–coated integrating sphere, to prevent absorption spectra distortion due to light scattering by the nanowires. The measurements of UV-vis-NIR absorption spectra were carried out in a 1-cm path-length quartz optical cell. The polymer nanowire solution was dispersed in methanol (spectroscopy-grade), and ultrasonication was applied for 10–20 secs before the absorption measurements. A field-emission scanning electron microscope (FE-SEM; sigma, Carl Zeiss, USA) and an atomic force microscope (AFM; XE-100, PSIA, South Korea) were used to observe the morphologies of the nanostructures. Structures of the TIIG-Bz assemblies with 2-BBA were analyzed in detail using PXRD and GIXD experiments at a synchrotron facility (PLS-II 6D UNIST-PAL beamlines at Pohang Accelerator Laboratory, South Korea). For PXRD measurements, dry samples of TIIG-Bz and TIIG-Bz/2-BBA assemblies were placed into polyimide capillary tubes and continuously rotated during the measurements. For GIXD measurements, methanol dispersions of the assemblies were dropped onto slices of silicon wafers and dried. The molecular spacing and packing orientation relative to the substrates were characterized using GIXD profiles. XPS was carried out using an AXIS Ultra DLD instrument (Kratos, U.K.) in an advanced *in-situ* surface analysis system (AISAS; KBSI, Korea) operating at a base pressure of 1.6 × 10^−10^ mbar at 300 K with a monochromatic Al Kα line at 1486.69 eV. The samples were prepared on a Si wafer substrate, or a Si wafer substrate coated with a 140-nm-thick gold layer, which were electrically grounded to the sample stage. The binding energy scales were calibrated by the C 1 s core level position at 284.8 eV as an internal reference and the Fermi edge of a gold standard. Survey and narrow spectrum scans were obtained with analyzer pass energies of 160 and 40 eV, respectively at 150 W. In order to separate the chemical bonding states in the spectra, the spectral line shape was simulated using Casa XPS software using a Shirley background and a GL (30) line shape (70% Gaussian, 30% Lorentzian).

## Electronic supplementary material

Supplementary Information

## Data Availability

All data generated or analysed during this study are included in this published article (and its Supplementary Information files). The datasets generated and analysed during the current study are available from the corresponding author on reasonable request.
