# Supplementary material for: Thienoisoindigo-Based Semiconductor Nanowires Assembled with 2-Bromobenzaldehyde via Both Halogen and Chalcogen Bonding
Source: Sci Rep. 2018 Sep 27;8:14448. doi: 10.1038/s41598-018-32486-z (PMC6160462; doi:10.1038/s41598-018-32486-z)
Supplement: Supplementary file 1 — Supplementary Information [file 41598_2018_32486_MOESM1_ESM.docx]

Supporting Information

**Thienoisoindigo-Based Semiconductor Nanowires Assembled with 2-Bromobenzaldehyde via Both Halogen and Chalcogen Bonding**

**Juran Noh^1,#^, Sungwoo Jung^2,#^, Dong Geon Koo^1,#^, Gyoungsik Kim^2^, Kyoung Soon Choi^3^, JaeHong Park^4^, Tae Joo Shin^5^, Changduk Yang^2,+,^ and Juhyun Park^1,*^**

^1^School of Chemical Engineering and Materials Science, Chung-Ang University (CAU), Seoul 06974, Republic of Korea

^2^Department of Energy Engineering, School of Energy and Chemical Engineering, Low Dimensional Carbon Materials Center, Ulsan National Institute of Science and Technology (UNIST), Ulsan 44919,

^3^Advanced Nano-Surface Research Group, Korea Basic Science Institute (KBSI), Daejeon 34133, Republic of Korea

^4^Department of Molecular Engineering, Kyoto University, Katsura, Nishikyo-ku, Kyoto 615-8510, Japan

^5^UNIST Central Research Facilities & School of Natural Science, Ulsan National Institute of Science and Technology (UNIST), Ulsan 44919, Republic of Korea.

^*^jpark@cau.ac.kr

^+^yang@unist.ac.kr


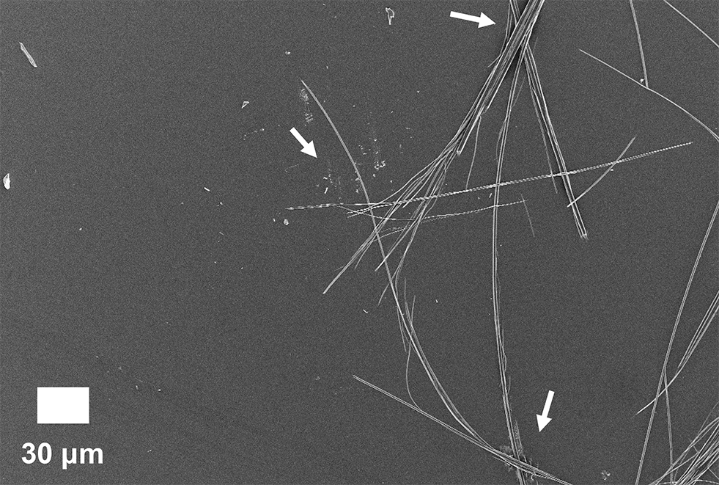


**Figure S1**. FE-SEM images of nascent TIIG-Bz wire assembly.


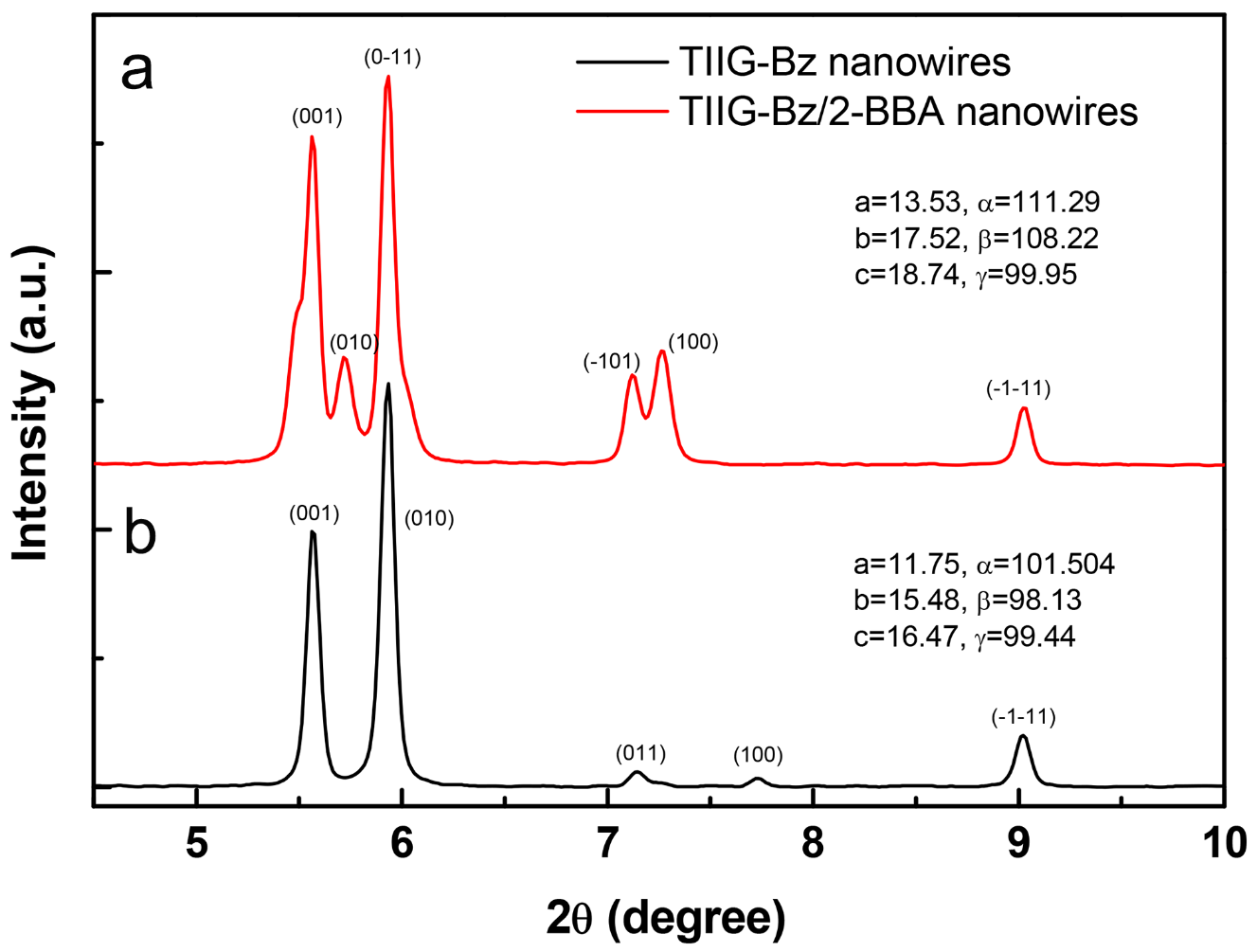


**Figure S2**. 1D PXRD profiles of (a) TIIG-Bz/2-BBA nanowires and (b) TIIG-Bz nanomaterial below 2θ = 10°.

**Table S1**. Representative crystallographic data of TIIG-Bz and TIIG-Bz/2-BBA assemblies.

| Samples | Crystal  plane | q  (Å^-1^) | d-spacing  (Å) | FWHM  (Å^-1^) | L_c_  (nm) |
| --- | --- | --- | --- | --- | --- |
| TIIG-Bz | (001) | 0.3962 | 15.859 | 0.004519 | 139.0257 |
|  | (010) | 0.4219 | 14.889 | 0.004756 | 132.1196 |
|  | (100) | 0.5499 | 11.427 | 0.005546 | 113.2908 |
| TIIG-Bz/2-BBA | (001) | 0.3961 | 15.863 | 0.005385 | 116.6772 |
|  | (010) | 0.4072 | 15.431 | 0.005672 | 110.7810 |
|  | (100) | 0.5168 | 12.157 | 0.006461 | 97.2449 |


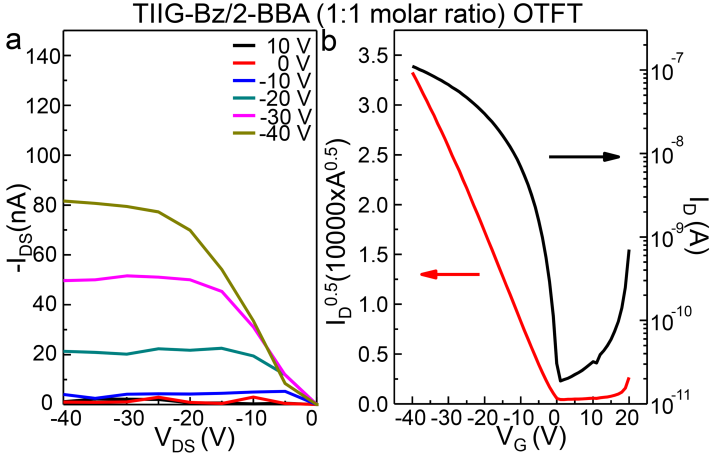

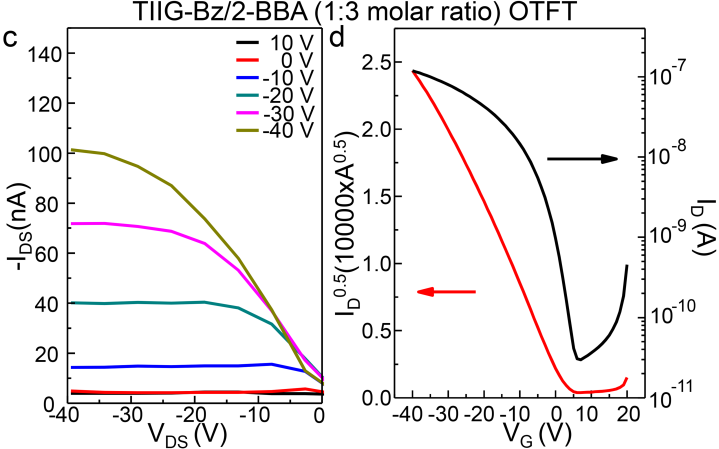

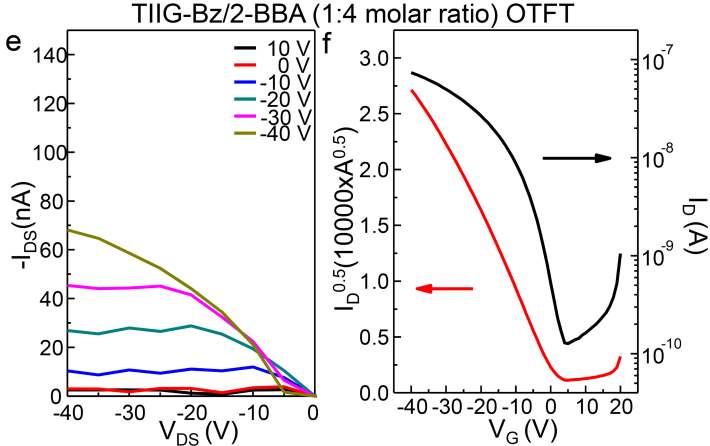

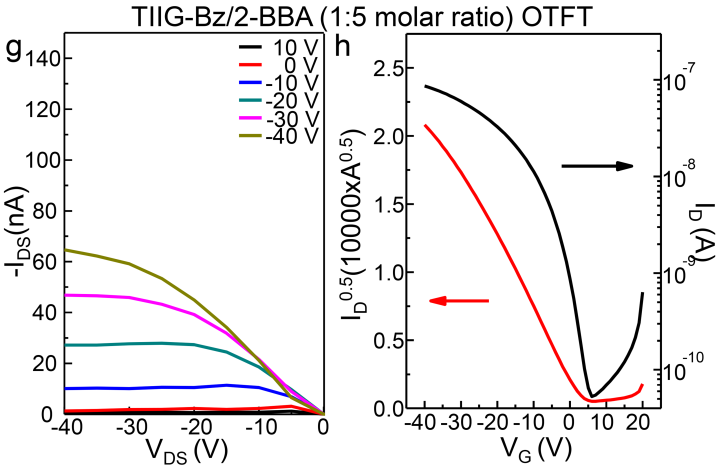


**Figure S3**. (a) Output and (b) transfer characteristics of OFET devices using thin films of TIIG-Bz/2-BBA at a 1:1 molar mixing ratio, (c) and (d) those at a 1:3 molar mixing ratio, (e) and (f) those at a 1:4 molar mixing ratio, and (g) and (h) those at a 1:5 molar mixing ratio.


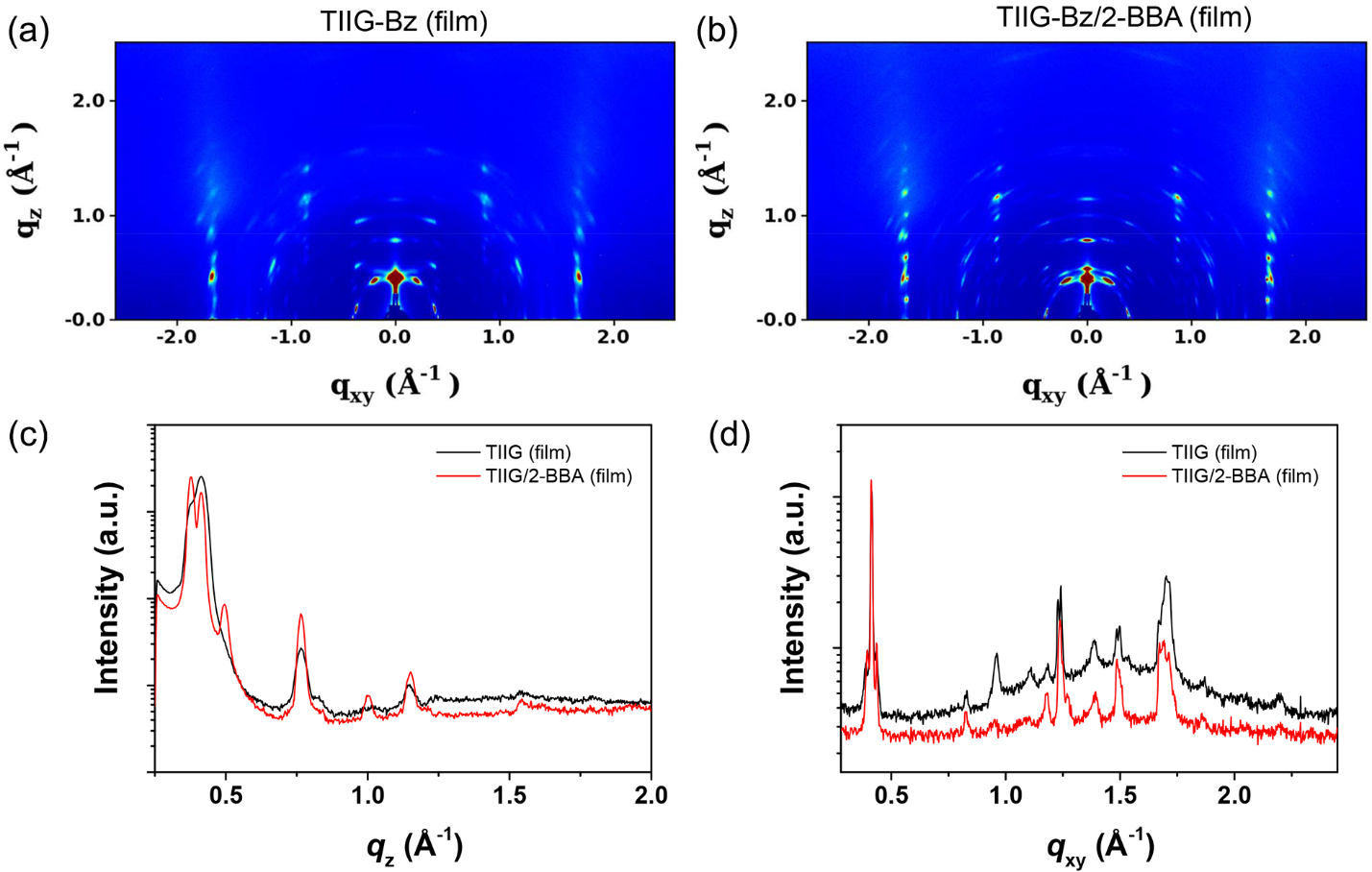


**Figure S4**. 2D GIXD patterns of (a) TIIG-Bz film and (b) TIIG-Bz/2-BBA film at a 1:2 mixing ratio, and corresponding GIXD line cuts along the (c) out-of-plane and (d) in-plane directions.
